# Calcium pyrophosphate dihydrate crystal deposition disease of the spinal dura mater: a case report

**DOI:** 10.1259/bjrcr.20170049

**Published:** 2017-10-07

**Authors:** Noriaki Wada, Koji Yamashita, Akio Hiwatashi, Osamu Togao, Ryotaro Kamei, Daichi Momosaka, Yasuhiro Maeda, Takuya Matsushita, Ryo Yamasaki, Keiichiro Iida, Yuichi Yamada, Jun-ichi Kira, Hiroshi Honda

**Affiliations:** ^1^Departments of Clinical Radiology, Graduate School of Medical Sciences, Kyushu University Hospital, Fukuoka, Japan; ^2^Department of Neurology, Neurological Institute, Graduate School of Medical Sciences, Kyushu University Hospital, Fukuoka, Japan; ^3^Department of Orthopaedic Surgery, Graduate School of Medical Sciences, Kyushu University Hospital, Fukuoka, Japan; ^4^Department of Anatomic Pathology, Pathological Sciences, Graduate School of Medical Sciences, Kyushu University Hospital, Fukuoka, Japan

## Abstract

Calcium pyrophosphate dihydrate (CPPD) crystal deposition disease is characterized by the accumulation of CPPD crystal in articular and periarticular tissues, but CPPD crystal deposition along the spinal dura mater has not been previously reported. We report a 54-year-old male presenting with progressive neck pain and numbness of the extremities. CT showed diffuse dorsal epidural calcification from C3-T6 which resulted in spinal canal stenosis. On MR imaging, the lesion was hypointense on both *T*_1_ and *T*_2_ weighted images. From these findings, CPPD crystal deposition in the ligamentum flavum was suspected preoperatively. Biopsy at the level of C5-6 were performed to confirm the diagnosis. Perioperative and histopathological findings revealed that CPPD crystals were deposited along the dorsal dura mater, not in the ligamentum flavum. We firstly report the CT and MR imaging features of a possible new concept in the differential diagnosis of CPPD crystal deposition disease.

## Background

Calcium pyrophosphate dihydrate (CPPD) crystal deposition disease is characterized by the accumulation of CPPD crystals in articular and periarticular tissues.^1–3^ CPPD crystal deposition can present with acute arthritis or chronic arthropathy with structural changes of osteoarthritis. The disease can also be asymptomatic.^1,4^ The most commonly affected joints are knees, followed by wrists, hands, pelvis, symphysis pubis and hips. Although CPPD crystal deposition disease of the spine is less common, it can involve the ligamentum flavum; longitudinal, supraspinous and interspinous ligaments; the intervertebral discs, and the sacroiliac and apophyseal joints.^2^ However, CPPD crystal deposition along the dorsal spinal dura mater has not been previously reported. We herein describe a case of diffuse calcification in the posterior epidural space at C3-T6 mimicking previously reported CPPD crystal deposition in the ligamentum flavum.^5^ Pathological examination after biopsy proved the lesion to be CPPD crystal deposition along the dura mater.

## Case report

**Figure 1.**
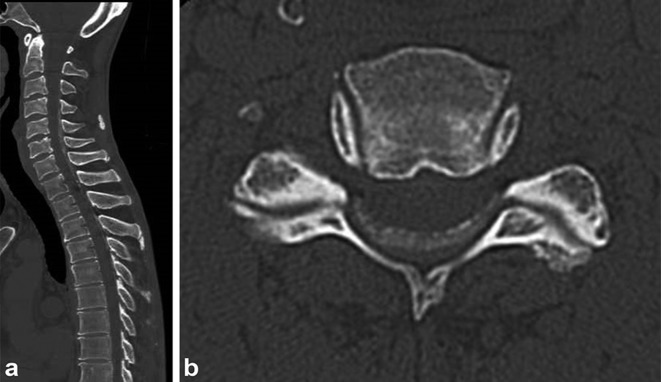
(a) Preoperative CT of the cervicothoracic spine. Sagittal CT shows diffuse epidural calcification at C3-T6. (b) Axial CT of the C5/6 level reveals crescent-shaped mass which has a well-defined border along the vertebral arch.

**Figure 2.**
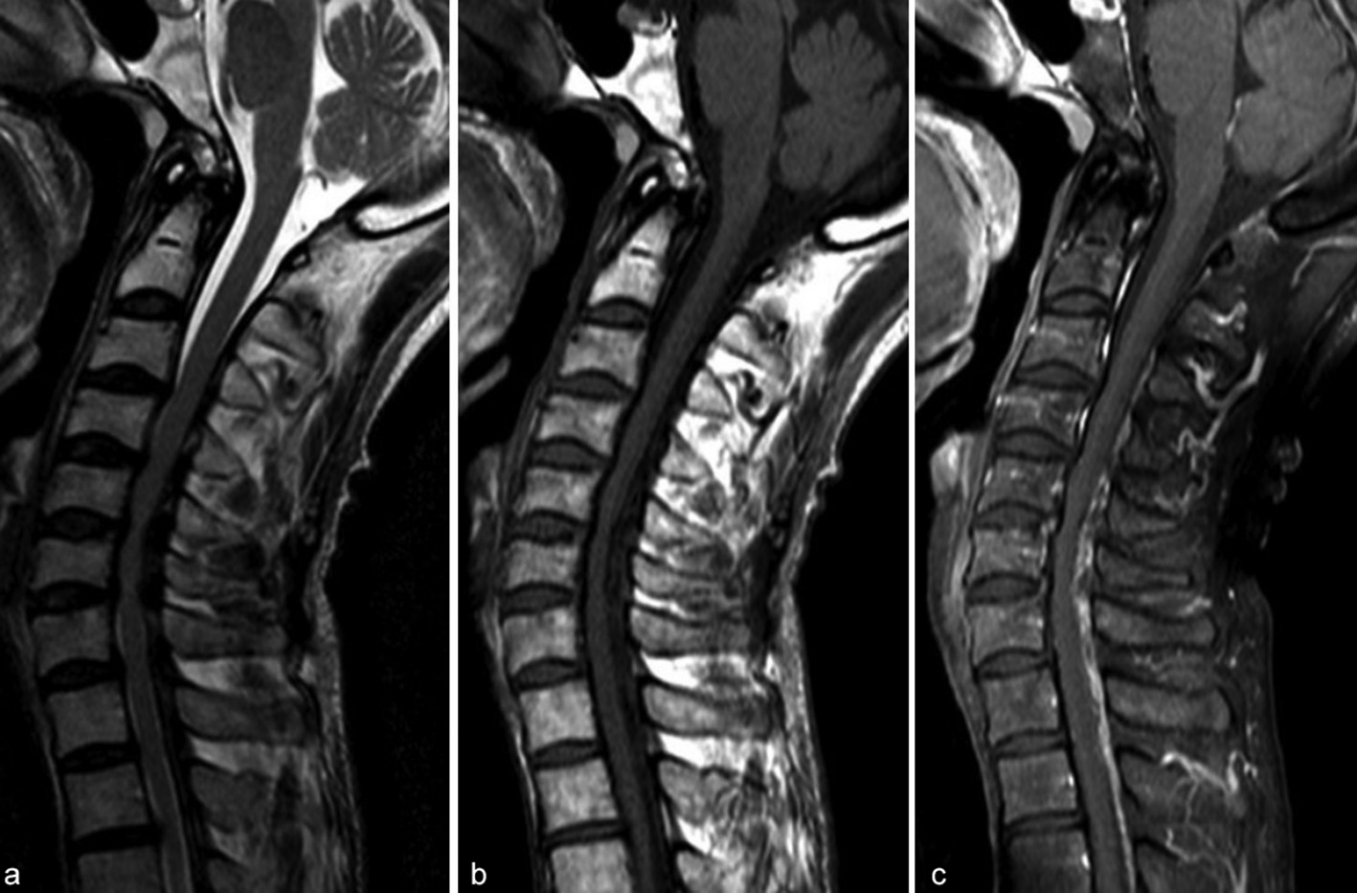
Preoperative MR imaging of the cervical spine. The lesion shows hypointensity on both (a) *T*_2_ and (b) *T*_1 _weighted images which causes spinal canal stenosis. (c) Sagittal post-contrast *T*_1_ weighted imaging demonstrates heterogeneous enhancement.

**Figure 3.**
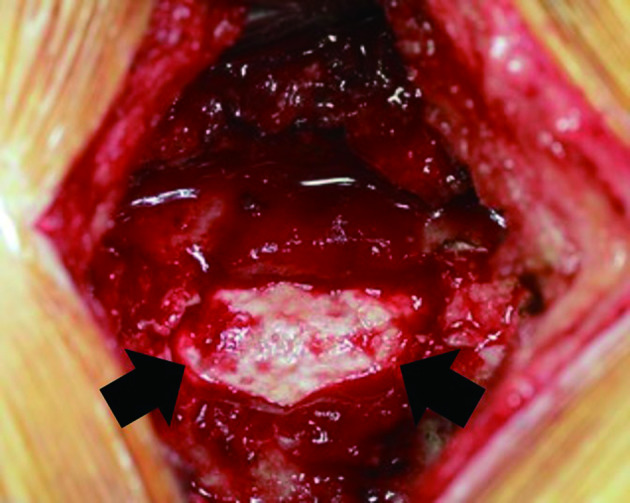
Perioperative photograph. Grayish-white, solid calcified mass (arrows) is found just beneath the ligamentum flavum (not shown).

**Figure 4.**
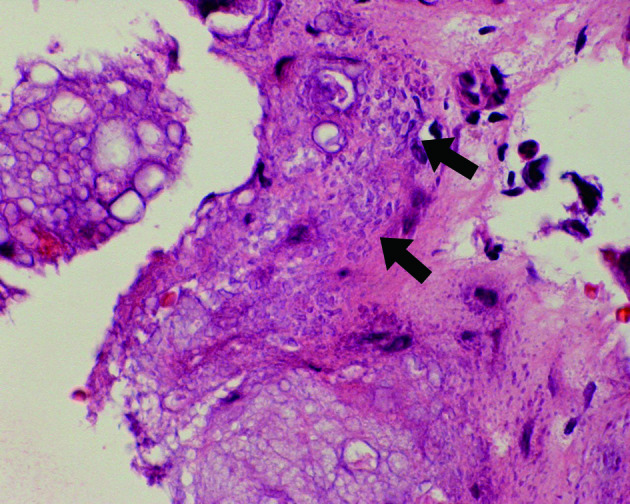
Photomicrograph of biopsy specimen (haematoxylin and eosin stain, original magnification ×100). Numerous granular calcium crystals (arrows) are deposited within dense fibrous tissue with myxoid change.

A 54-year-old male presented with progressively worsening neck pain and numbness of the left upper extremity and the left face for 2 weeks. He had no history of recent head or neck injuries. He was followed closely without further treatment because of his fluctuating subjective symptoms. However, the symptoms gradually worsened, and he visited our hospital for intensive examination and treatment 2 months after the onset. Neurological examination revealed a mildly spastic gait and exaggerated deep tendon reflexes in the bilateral upper and lower extremities. He also had mild dysuria and constipation. Muscle strength and vibration sense were normal, and no Babinski sign was present. Peripheral blood examination on admission revealed no significant abnormality. Cerebrospinal fluid examination was within normal limits except for slightly elevated protein (65 mg dl^–1^; normal, 10–40 mg dl^–1^). Unenhanced CT of the cervicothoracic spine showed diffuse dorsal epidural calcification at C3-T6. The lesion presented as a crescent shape along the spinal dura mater and had a well-defined border along the vertebral arch (Figure 1). On MR imaging, the lesion appeared as a hypointense mass on both pre-contrast *T*_1_ and *T*_2_ weighted images. The lesion displayed heterogeneous enhancement, and caused cervical spinal canal stenosis (Figure 2). Head CT and MR imaging revealed no significant abnormality. Based on these findings, CPPD crystal deposition disease of the ligamentum flavum was suspected. C5/6 laminectomy to biopsy for confirmation was performed. Perioperative findings revealed an intact ligamentum flavum. A solid, grayish-white lesion was found just beneath the ligamentum flavum (Figure 3). Histopathological examination of the specimen demonstrated numerous granular calcium crystals within dense fibrous tissue with myxoid change (Figure 4). Grocott, periodic acid-Schiff and Ziehl-Neelsen stains showed no obvious source of infection (*e.g*. fungal or tubercular), and there was no evidence of malignancy. These features confirmed the diagnosis of CPPD crystal deposition disease along the spinal dura mater. The patient’s neurological symptoms during the postoperative course was uneventful, and follow-up CT performed 4 months later revealed no regrowth of the calcified lesion.

## Discussion

To the best of our knowledge, CPPD crystal deposition along the spinal dura mater has not been previously reported. CPPD crystal deposition disease is found in 9.6% of individuals older than 50 years, and this incidence increases with age.^3^ Both aging and osteoarthritis are independently associated with CPPD crystal deposition. An increased risk of CPPD crystal deposition disease, as a result of previous joint injury and metabolic diseases such as haemochromatosis, hyperparathyroidism, and hypomagnesemia has been reported.^1,4^ CT is useful to identify CPPD crystal deposits and usually nicely demonstrates the topography of the calcification and associated bone changes. On MR imaging, CPPD crystal deposition disease manifest as a predominately hypointense area on pre-contrast *T*_1_ and *T*_2_ weighted images as in the present case.^5^ Post-contrast T1 weighted imaging demonstrates heterogeneous enhancement,^5^ which may represent fibrous tissue or inflammation on pathological examination. MR imaging is also useful to assess spinal cord compression or myelopathy.^6^

The differential diagnosis of diffuse epidural calcification with hypointensity in the spine on pre-contrast MR imaging includes CPPD crystal deposition disease in the ligamentum flavum (CLF), ossification of the ligamentum flavum, amyloidosis and hyperparathyroidism associated with chronic renal failure or alone and tuberculosis. CLF predominantly affects elderly women and frequently occurs in the lower part of the cervical spine.^5–8^ CT shows either nodular-type calcification which appears to partially contact the vertebral arch, or diffuse-type calcification close to the vertebral arch^5–8^ in which calcified granules are deposited within the degenerated ligamentous fibres histopathologically.^5–7^ Multiple levels can be affected, and CPPD crystal deposition extending maximally from C2-T3 has been reported.^5^ Some cases with CPPD crystal deposition along the spinal dura mater may have been previously confused with CLF. Diffuse-type CLF has a pattern of distribution of epidural calcification similar to the present case. However, in our case, diffuse calcification had no continuity with the vertebral arch on CT. This finding may suggest an intact ligamentum flavum and provide helpful information in discrimination the two. Ossification of the ligamentum flavum is frequently located in the lower thoracic spine and predominantly affects middle-aged men.^9,10^ CT shows a V-shaped ossification,^9^ and mature bone is shown to have formed within the ligament histopathologically.^7,9^ Amyloid consists of relatively insoluble fibrils consisting of β2-microglobulin and its deposition can occur in a systemic or localized pattern.^10^ Primary solitary amyloidoma of the spine is extremely rare. Spinal amyloidosis was characterized by bone destruction and soft tissue extension. Hyperparathyroidism associated with chronic renal failure or alone and tuberculosis are common causes of dural calcification. The blood test showed renal function and calcium as well as intact parathyroid hormone were normal. The Quantiferon-TB Gold test was negative in our case.

In conclusion, we believe we are the first to report CPPD crystal deposition disease along the spinal dura mater. We observed a multilevel, crescent-shaped, diffusely calcified lesion with a well-defined border with the vertebral arch, which may be characteristic of CPPD crystal deposition along the dorsal spinal dura mater, rather than in the ligamentum flavum.

## Learning points

1. This is the first report describing a patient with calcium pyrophosphate dihydrate (CPPD) crystal deposition along spinal dura mater.
2. Preoperatively, CPPD crystal deposition disease in ligamentum flavum was suspected.
3. CT showed multilevel, crescent-shaped, diffusely calcified lesion with well-defined border with the vertebral arch, which may be characteristic of CPPD crystal deposition along the dorsal spinal dura mater, rather than in the ligamentum flavum.

## Consent

Written informed consent for the case to be published (including images, case history and data) was obtained from the patient(s) for publication of this case report, including accompanying images.

## Acknowledgement

 This work was supported by Grant-in-Aid for Scientific Research (C) Grant Number 17K10365. 
